# Whole-Genome Sequence of the Novel Rubrobacter taiwanensis Strain Yellowstone, Isolated from Yellowstone National Park

**DOI:** 10.1128/MRA.00287-19

**Published:** 2019-04-18

**Authors:** Shawn Freed, Robert F. Ramaley, John A. Kyndt

**Affiliations:** a College of Science and Technology, Bellevue University, Bellevue, Nebraska, USA; b Department of Biochemistry and Molecular Biology, UNMC, Omaha, Nebraska, USA; University of Southern California

## Abstract

The family *Rubrobacteraceae* is often represented by its thermophilic and radiotolerant species. Rubrobacter radiotolerans and Rubrobacter xylanophilus have been extensively studied, contributing to defining the characteristics of the family. We have now sequenced the genome of a Rubrobacter taiwanensis strain, isolated from Yellowstone National Park, to further characterize the family.

## Abstract (precis)

The family *Rubrobacteraceae* is often represented by its thermophilic and radiotolerant species. Rubrobacter radiotolerans and Rubrobacter xylanophilus have been extensively studied, contributing to defining the characteristics of the family.

## ANNOUNCEMENT

The key feature of *Rubrobacter* species is their thermophilic behavior. This was first observed in isolates of R. xylanophilus and R. radiotolerans (1, 2) and has since been documented in two newer species, R. calidifluminis and R. naiadicus (3). They commonly exhibit halotolerance and have been found to live in hot springs around the world, but their most unique characteristic is their high tolerance to gamma radiation. This has been hypothesized to be a by-product of defense against desiccation, similar to that in the radiation-tolerant genus *Deinococcus*, which is found in high gamma radiation environments (2, 4). The documented species in this family are recorded as having modest genome sizes (around 2 to 3 Mbp) and commonly contain genes for reduction of nitrates to nitrite, as well as cytochrome oxidase and catalase (1, 4). A more recent study suggests the presence of proteins involved in chlorophyll biosynthesis, suggesting a photosynthetic pathway (5).

A pink isolate was obtained from the Mushroom Hot Spring runoff in Yellowstone National Park, with a sampling point temperature of 50°C (lat 44.5387, long –110.798). The sample was obtained from the microbial mat and its surrounding water. The isolate was purified by streaking on solid *Thermus* medium with 0.3% tryptone and 0.3% yeast extract (6). A partial 16S rRNA gene was sequenced (530 bp) by MIDI Labs (Newark, DE). A BLAST (NCBI) comparison showed 99% and 100% similarity with Rubrobacter taiwanensis LS286 and LS293, respectively, 96% similarity with Rubrobacter spartanus, 95% similarity with Rubrobacter xylanophilus, and 94% similarity with Rubrobacter radiotolerans. No genome sequence is currently available for either of the closely related *R. taiwanensis* strains, which were isolated previously from hot springs in Taiwan (7). We have sequenced the genome of this new isolate to further characterize the species identity.

We isolated DNA of our *R. taiwanensis* isolate from living culture grown on *Thermus* medium with 0.3% yeast extract and 0.15% fructose and ribose. Genomic DNA was isolated using the GeneJET DNA purification kit (Thermo Scientific) to perform a genome comparison and explore the similarities and differences from more extensively studied species. Utilizing Qubit and NanoDrop technologies, we determined the quality and quantity of DNA, showing a 260/280 ratio of 1.79. The genome was sequenced by an Illumina MiniSeq system using 500 μl of a 1.8 pM library. Paired-end (2 × 150-bp) sequencing generated 3,883,502 reads and 330 Mbp. Quality control of the reads was performed using FastQC within BaseSpace version 1.0.0 (Illumina) using a k-mer size of 5 and contamination filtering. We performed *de novo* assembly using Velvet version 1.2.10 (8) through BaseSpace using a minimum k-mer size of 21 and a maximum k-mer size of 121 and including reverse complement reads. This assembly yielded 53 contigs (≥1,000 bp), the largest being 350,459 bp, and an *N*_50_ value of 112,895 bp. The GC content was 67.2%. We then annotated the genome sequence using Rapid Annotations using Subsystems Technology (RAST) version 2.0 (9), using the RASTk default annotation pipeline, including the automated error fix and gap backfill settings. This showed our strain to be 3,035,128 bp long with 3,170 coding sequences and 48 RNAs identified.

A JSpecies comparison (10) of average percentage nucleotide identities (ANI) between this *R. taiwanensis* genome and other published *Rubrobacteraceae* genomes gave the following percentages: 74.2% (R. xylanophilus DSM9941), 71.4% (R. aplysinae), 71.0% (R. radiotolerans RSPS-4), and 69.6% (R. indicoceanis). Thus, *R. taiwanensis* appears to be approximately equidistant to the other four *Rubrobacter* species that have been sequenced. They are all more distant from Kineococcus radiotolerans, with an ANI of 65.0%. The *R. taiwanensis* ANI numbers are clearly below the proposed 95% cutoff for the genome definition of a species (10), suggesting its own species identity.

### Data availability.

This whole-genome shotgun project has been deposited at DDBJ/ENA/GenBank under the accession number SKBU00000000. The version described in this paper is version SKBU01000000. The raw sequencing reads have been submitted to the SRA, and the corresponding accession number is SRR8670886.

## References

[1] Carreto L, Moore E, Nobre MF, Wait R, Riley PW, Sharp RJ, da Costa MS 1996 *Rubrobacter xylanophilus* sp. nov., a new thermophilic species isolated from a thermally polluted effluent. Int J Syst Evol Microbiol 46:460–465. doi:10.1099/00207713-46-2-460.

[2] Ferreira AC, Nobre MF, Moore E, Rainey FA, Battista JR, da Costa MS 1999 Characterization and radiation resistance of new isolates of *Rubrobacter radiotolerans* and *Rubrobacter xylanophilus*. Extremophiles 3:235–238. doi:10.1007/s007920050121. 10591012

[3] Albuquerque L, Johnson MM, Schumann P, Rainey FA, da Costa MS 2014 Description of two new thermophilic species of the genus *Rubrobacter*, *Rubrobacter calidifluminis* sp. nov. and *Rubrobacter naiadicus* sp. nov., and emended description of the genus *Rubrobacter* and the species *Rubrobacter bracarensis*. Syst Appl Microbiol 37:235–243. doi:10.1016/j.syapm.2014.03.001. 24780859

[4] Egas C, Barroso C, Froufe HJC, Pacheco J, Albuquerque L, da Costa MS 2014 Complete genome sequence of the radiation-resistant bacterium *Rubrobacter radiotolerans* RSPS-4. Stand Genomic Sci 9:1062–1075. doi:10.4056/sigs.5661021. 25197483 PMC4148983

[5] Gupta RS, Khadka B 2016 Evidence for the presence of key chlorophyll-biosynthesis-related proteins in the genus *Rubrobacter* (phylum Actinobacteria) and its implications for the evolution and origin of photosynthesis. Photosynth Res 127:201–218. doi:10.1007/s11120-015-0177-y. 26174026

[6] Ramaley RF, Hixson J 1970 Isolation of a nonpigmented, thermophilic bacterium similar to *Thermus aquaticus*. J Bacteriol 103:527–528. 5432016 10.1128/jb.103.2.527-528.1970 PMC248117

[7] Chen M-Y, Wu S-H, Lin G-H, Lu C-P, Lin Y-T, Chang W-C, Tsay S-S 2004 *Rubrobacter taiwanenesis* sp. nov., a novel thermophilic, radiation-resistant species isolated from hot springs. Int J Syst Evol Microbiol 54:1849–1855. doi:10.1099/ijs.0.63109-0. 15388754

[8] Zerbino DR, Birney E 2008 Velvet: algorithms for *de novo* short read assembly using de Bruijn graphs. Genome Res 18:821–829. doi:10.1101/gr.074492.107. 18349386 PMC2336801

[9] Aziz RK, Bartels D, Best AA, DeJongh M, Disz T, Edwards RA, Formsma K, Gerdes S, Glass EM, Kubal M, Meyer F, Olsen GJ, Olson R, Osterman AL, Overbeek RA, McNeil LK, Paarmann D, Paczian T, Parrello B, Pusch GD, Reich C, Stevens R, Vassieva O, Vonstein V, Wilke A, Zagnitko O 2008 The RAST server: Rapid Annotations using Subsystems Technology. BMC Genomics 9:75. doi:10.1186/1471-2164-9-75. 18261238 PMC2265698

[10] Richter M, Rosselló-Móra R, Glöckner FO, Peplies J 2016 JSpeciesWS: a Web server for prokaryotic species circumscription based on pairwise genome comparison. Bioinformatics 32:929–931. doi:10.1093/bioinformatics/btv681. 26576653 PMC5939971

